# Characterizing *Escherichia coli* carrying plasmid-mediated AmpC β-lactamases to optimize detection in a diagnostic laboratory setting

**DOI:** 10.1128/spectrum.00933-24

**Published:** 2024-11-07

**Authors:** Jason Teng, Luke Walters, Lex E. X. Leong, Kija Smith, Malissa Amato, Xiao Chen, Mark Turra, Morgyn S. Warner, Lito E. Papanicolas

**Affiliations:** 1 Department of Microbiology and Infectious Diseases, Flinders Medical Centre, Bedford Park, South Australia, Australia; 2 Microbiology and Infectious Diseases, SA Pathology, Adelaide, South Australia, Australia; 3 Microbiology and Infectious Diseases, SA Pathology, Royal Adelaide Hospital, Adelaide, South Australia, Australia; 4 College of Medicine and Public Health, Flinders University, Bedford Park, South Australia, Australia; Seton Hall University, South Orange, New Jersey, USA

**Keywords:** AmpC, whole-genome sequencing, antimicrobial resistance, *Escherichia coli*, PCR

## Abstract

Plasmid-mediated AmpC β-lactamases are a cause of acquired cephalosporin resistance in Gram-negative bacteria. However, consensus regarding the optimal detection method is yet to be achieved and varies depending on local epidemiology and laboratory capacity. We determined the acquired genotypic resistance mechanisms of 250 *Escherichia coli* isolates with a positive AmpC screen, defined as cefoxitin MIC ≥ 8 mg/L and a positive AmpC double- disc diffusion test, using in-house designed high-resolution melt PCR, detecting plasmid-acquired genes from the CIT and DHA families. A proportion of these isolates (*n* = 170, 68%) underwent further genotypic characterization using whole- genome sequencing (WGS). Of 250 isolates with a positive screening test, 72 (28.8%) were determined to carry an acquired AmpC gene. There was 100% concordance between PCR and WGS in the identification of acquired AmpC genes. The phenotypic criteria were then assessed to determine their utility in predicting acquired AmpC gene carriage. Criteria 1 (cefoxitin MIC > 8 mg/L plus ceftriaxone MIC > 1 mg/L and/or ceftazidime MIC > 1 mg/L) yielded a sensitivity of 93.1% and a specificity of 47.8%. Criteria 2 (cefoxitin MIC > 16 mg/L plus ceftriaxone MIC > 4 mg/L) had a sensitivity of 33.3% and a specificity of 98.9%. DHA genes, whose expression may be induced following antibiotic exposure, were present in 19% of isolates testing susceptible to ceftriaxone (MIC ≤ 1 mg/L) and were significantly more likely than CIT genes to be detected in susceptible isolates (*P* < 0.0001). These findings highlight the importance of using genotypic methods to detect acquired AmpC resistance in *E. coli* isolates that meet phenotypic screening criteria.

**IMPORTANCE:**

Detection of transmissible AmpC resistance remains a challenging problem for diagnostic laboratories, especially in *Escherichia coli* where the expression of its intrinsic AmpC gene can result in phenotypic resistance patterns indistinguishable from plasmid-mediated resistance. In conjunction with whole- genome sequencing (WGS), we describe the development and performance of a novel melt-curve PCR to identify the two most prevalent plasmid-mediated AmpC gene families: CIT and DHA. We then describe phenotypic testing algorithms that incorporate this PCR and can differentiate these from non-acquired resistance in *E. coli*. It is important to distinguish these, not only to spare patients from unnecessarily being treated with infection control precautions, but also to identify plasmid-mediated genes, especially of the DHA family, that have been associated with inducible drug resistance to third- generation cephalosporins.

## INTRODUCTION

The escalating threat of antimicrobial resistance represents a major worldwide health concern with an estimated 4.95 million deaths in 2019 associated with bacterial resistance according to statistical modeling (1). Over recent decades, AmpC β-lactamases have emerged as a widely distributed mechanism of Gram-negative resistance (2). Effective detection of AmpC enzymes is of critical importance as it equips clinicians with essential information for optimal antimicrobial selection and directs infection-control measures (3).

Broadly speaking, AmpC β-lactamase resistance in Enterobacterales occurs either through the expression of intrinsic resistance of an AmpC gene carried in the organism’s chromosome or via acquired resistance, generally through the acquisition of an AmpC gene on an extra-chromosomal plasmid. Regardless of the mechanism, AmpC gene expression results in a broad range of β-lactam resistance including to penicillins, most cephalosporins, cephamycins, and monobactams. They are not affected by traditional β-lactamase inhibitors (2), such as clavulanic acid, but are inhibited by newer generation inhibitors such as avibactam and vaborbactam (4) and are also suppressed in the presence of cloxacillin and 3-aminophenylboronic acid (4). In certain species such as *Enterobacter cloacae* and *Citrobacter freundii,* chromosomal resistance is usually expressed. In these species, AmpC gene expression is inducible through β-lactam antibiotic use although mutations in regulatory proteins can result in constitutive expression and high-level phenotypic resistance that is de-coupled from antibiotic induction (4).

Acquired plasmid-mediated AmpC β-lactamase detection is particularly important from an infection control perspective, given the potential for horizontal transmission (5). Plasmid-mediated AmpC genes are classified into families based on the chromosomally encoded genes from which they are thought to have originated. These families include DHA, CIT, FOX, MOX, ACC, and EBC (2). For example, AmpC β-lactamase genes *bla*_CMY-2_, *bla*_BIL-1,_ and *bla*_LAT-1_ are all related to the *Citrobacter freundii* chromosomal AmpC gene and are, therefore, part of the CIT family (6). Notably, *bla*_DHA-1_, *bla*_DHA-2_, *bla*_CMY-13,_ and *bla*_ACT-1_ genes are inducible, as are many chromosomally carried AmpC genes, through linkage to the regulatory protein AmpR (2). As with chromosomally carried inducible AmpC genes, high-level resistance may not be detectable phenotypically prior to the induction of the enzyme in the presence of beta-lactam therapy (7).

The detection of acquired AmpC β-lactamases in *Escherichia coli* is particularly challenging because *E. coli* carries a chromosomal AmpC gene that is usually not expressed or inducible due to the absence of AmpR (2). However, mutations in the promoter or attenuator regions can cause low- level constitutive hyperexpression of AmpC in *E. coli*, resulting in variable degrees of phenotypic resistance to cephalosporins (4). This chromosomal gene expression can be difficult to differentiate from acquired mechanisms of resistance with phenotypic testing alone (8). However, since *E. coli* intrinsic AmpC is neither transmissible nor inducible, its expression does not have the infection control or clinical treatment implications associated with acquired AmpC genes.

While standardized methods of extended spectrum β-lactamase (ESBL) testing have been developed, the optimal approach for AmpC detection remains undetermined (3). At present, a wide variety of approaches are employed by different laboratories. There are no recommendations from The Clinical and Laboratory Standards Institute (CLSI) on AmpC detection (9). Currently, the European Committee on Antimicrobial Susceptibility Testing (EUCAST) suggest screening with the criteria of cefoxitin resistance combined with ceftazidime and/or cefotaxime resistance, followed by a confirmatory test utilizing cloxacillin or boronic acid synergy, and proceeding to polymerase chain reaction (PCR) to detect acquired genes if the organism is *E. coli* or *Shigella* (10).

Molecular tests such as PCR are important ways to identify acquired AmpC genes in *E. coli* where phenotypic tests cannot reliably exclude their presence (6, 10). These have the added benefit of distinguishing between families of AmpC genes and, in the case of WGS, enable the identification of other acquired resistance genes. Multiplex PCR using primer sets for each of the AmpC families has been described and is considered the gold standard of plasmid-mediated AmpC detection (6). Some laboratories employ this method as a confirmatory test for all isolates with suspected AmpC production following an initial screening test (11). However, significant costs, labor, equipment requirements, and reagent availability may limit the use of this method. Likewise, the application of WGS is limited by these factors, as well as long turnaround times and requirements for technical skill and expertise in data interpretation.

Given the lack of standardized methods for the detection of plasmid-mediated AmpC genes, their exact prevalence is unknown (3). One study of ceftriaxone non-susceptible Enterobacterales isolates in the United States found that plasmid-mediated AmpC genes were expressed by 13.7% of all isolates and predominantly included *bla*_CMY-2_ (32%) and *bla*_DHA_ (30%) (12). In a study from Australia, New Zealand, and Singapore, plasmid-mediated AmpC β-lactamases were produced by 17% of ceftriaxone-resistant *E. coli* isolates, with *bla*_CMY-2_ representing over 90% (11). Similarly, another study from Australia found that *bla*_CMY-2_ and *bla*_DHA_ predominated accounting for 84.6% and 15.4% of plasmid-mediated AmpC genes, respectively (13). Our local epidemiology (see Materials and Methods) was concordant with this published data, and therefore, we designed a PCR assay targeting the CIT and DHA families to use as a confirmatory test within our laboratory, as described below.

The aims of this study were to (i) evaluate the performance of an in-house PCR assay for detecting DHA and CIT gene families, (ii) document the local epidemiology of acquired Amp-C resistance in *E. coli* using WGS, and (iii) evaluate ceftriaxone and ceftazidime MICs in *E. coli* isolates that harbor plasmid-mediated Amp-C genes compared to those testing negative in order to design the best laboratory work-flow to identify true-positive Amp-C acquired gene carrying isolates.

## MATERIALS AND METHODS

### Description of phenotypic criteria evaluated in this study

In our laboratory, *E. coli* isolates being tested for resistance undergo screening for AmpC using a double- disc diffusion method if flagged as possible AmpC using the criteria of a cefoxitin MIC ≥ 8 mg/L. Historically, such isolates proceeded to a complete multiplex PCR (6) for the detection of plasmid- mediated AmpC production. However, during the COVID-19 pandemic, the need to preserve PCR reagents and staffing for COVID-19 testing led to the laboratory adopting a purely phenotypic method to report AmpC detection. This method of phenotypic confirmation required isolates to demonstrate cefoxitin non-susceptibility in combination with ceftazidime and/or ceftriaxone non-susceptibility (Criteria 1) and was based on unpublished internal laboratory data indicating this method had a high sensitivity. Another set of criteria (Criteria 2) was based on a phenotypic confirmation method that internal laboratory testing suggested would have a high specificity. It required a cefoxitin MIC > 16 mg/L in addition to a ceftriaxone MIC > 4 mg/L (Table 1). The ceftriaxone MIC cut-off was set to >4 mg/L as this is the ceiling MIC on the antimicrobial susceptibility testing cartridges utilized by our core laboratory using the BD Phoenix instrument (see Antimicrobial susceptibility testing).

**TABLE 1:** Phenotypic screening and confirmatory criteria

Phenotypic screening method	
Screening criteria	Cefoxitin MIC ≥ 8 mg/L Followed by MASTDISC D68C AmpC test
**Phenotypic confirmatory method**	
Criteria 1	Cefoxitin non-susceptible (MIC > 8 mg/L) plus Ceftazidime non-susceptible (MIC > 1 mg/L) and/or Ceftriaxone non-susceptible (MIC > 1 mg/L)
Criteria 2	Cefoxitin R (MIC > 16 mg/L) and Ceftriaxone MIC > 4 mg/L

### Collection of isolates

Clinical isolates of *E. coli* were prospectively collected from a major Australian microbiology laboratory from July 2021 to March 2022. In addition, clinical isolates previously collected from December 2020 to May 2021 were retrospectively evaluated for inclusion in the study. These isolates represented 250 unique clinical episodes from 246 unique individuals. Isolates were selected on the basis of a positive AmpC screen, requiring both the detection of a cefoxitin MIC ≥ 8 mg/L and a positive AmpC test using MASTDISC D68C *Combi* AmpC ESBL Detection sets (Mast Group) (see Double-disc diffusion for AmpC detection). The identification of isolates was confirmed by MALDI-TOF (Bruker). Specimen type, organism and cefoxitin, ceftazidime, and ceftriaxone MICs were recorded for all isolates.

### Double- disc diffusion for AmpC detection

MASTDISC D68C *Combi* AmpC ESBL tests were performed and interpreted as per manufacturer instructions. This involved the preparation of a 0.5 McFarland standard saline suspension of isolate which was subsequently inoculated on Mueller-Hinton agar in accordance with the Kirby-Bauer disk diffusion susceptibility testing protocol. One disc of each of cefpodoxime (A), cefpodoxime/clavulanic acid (B), cefpodoxime/cloxacillin (C), and cefpodoxime/clavulanic acid/cloxacillin (D) was placed onto the agar. The discs were then incubated overnight at 35 ± 2°C. Zone sizes for all discs were measured. Isolates were considered positive for AmpC activity if both C–A and D– B were ≥5 mm. If B–A and D–C were ≥5 mm, the isolate was considered positive for ESBL activity, and if D –C was ≥5 mm but B –A was ≤5 mm, the isolate was considered positive for both ESBL and AmpC activity.

### Antimicrobial susceptibility testing

The susceptibility profile of each isolate to cefoxitin, ceftriaxone, and ceftazidime was evaluated. MICs were determined using automated antimicrobial susceptibility testing systems, including Phoenix (Becton Dickinson) or VITEK (bioMérieux) in accordance with manufacturer instructions. Interpretation of MICs was performed using EUCAST Clinical Breakpoint Tables (14). Isolates were evaluated against the proposed phenotypic confirmatory tests, Criteria 1 and Criteria 2 (Table 1).

### High- resolution melting PCR

In 2021, whole- genome sequencing (WGS) data were collected on 159 isolates from our laboratory to investigate the local prevalence of acquired AmpC genes, and we identified genes only from the DHA and CIT families. For this reason, we endeavored to develop a PCR test to specifically detect these two gene families. The high- resolution melting (HRM) PCR we developed was based on the multiplex assay described by Geyer and Hanson (15). A colony of each sample was suspended in 300 µL of MagNA Pure 96 External Lysis Buffer (Roche). A further 300 µL of distilled water was added to each tube. Two-hundred microliters of each sample and control was extracted using the MagNA Pure 96 System (Roche). Type-it HRM PCR Master Mix (Qiagen) was prepared in accordance with manufacturer instructions, containing Type-it HRM PCR Buffer with EvaGreen dye, dNTP mix, Q-Solution, and HotStarTaq Plus DNA Polymerase. CIT and DHA primer sequences were utilized. Automated PCR set-up was performed using the QIAgility system (Qiagen) with Hard-Shell 96-Well PCR plates (Bio-Rad). For each sample, 22 µL of Type-it HRM PCR Master Mix was combined with 3 µL of template. Sealed plates were spun and subsequently cycled on CFX96 Real-Time PCR System Bio-Rad Cycler for a total of 40 cycles. Results were exported to Bio-Rad CFX Maestro software. A Cq value ≤30 with a melt of <85.8 was reported as CIT, while a Cq value ≤30 with a melt of ≥85.8 was reported as DHA. The details of the development and validation of this in-house assay are provided in Supplemental methods.

### Whole- genome sequencing

Where available, pure isolates retrieved from storage at −80°C were re-cultured and underwent WGS. This resulted in the sequencing of 170 *E. coli* isolates. Due to the retrospective nature of part of this analysis, the remaining 80 had not been stored. Genomic DNA was extracted using QIASymphony DSP Viral/Pathogen Mini kit. Library preparation was performed with the Nextera XT Library Preparation Kit in accordance with the manufacturer instruction. Briefly, gDNA was enzymatically fragmented and barcoded with low cycle amplification (12-cycles). Amplicon libraries were quantified using Quant-IT dsDNA HS kit (Invitrogen, MA, USA) and LabChip DNA High Sensitivity Reagent kit (Revvity, MA, USA) on a LabChip GX Touch HT Nucleic Acid Analyzer system. Sequencing of libraries was subsequently performed on an Illumina NextSeq 550 platform with NextSeq 500/550 v2.5 kit (2 × 150 cycles, Illumina, CA, USA).

### Bioinformatic analysis

Read quality was assessed using seqtk (v1.3-r106), and taxonomic identification from raw sequencing reads was performed using Kraken2 (v2.1.2), a kmer-based approach for ultrafast assignment of taxonomy. Reads that passed the filter quality check will be used for *de novo* assembly using shovill (1.1.0), followed by contig filtering by removing contigs smaller than 500 bp. The filtered contigs were screened for antimicrobial resistance genes using ABRicate (1.0.1) against the Comprehensive Antibiotic Resistance Database (16) to detect known plasmid-mediated AmpC beta-lactamase genes (ACT-, ACC-, CMY-, DHA-, MIR-, MOX-).

### Statistical analysis

For each set of criteria, sensitivity, specificity, positive predictive value, and negative predictive value were calculated against the standard of multiplex PCR. A chi-square test was also performed for each set of criteria using a freely available on-line calculator (https://www.socscistatistics.com/tests/chisquare2/default2.aspx).

## RESULTS

### Description of clinical isolates

A total of 250 *E. coli* isolates were included in the study. Of these, 82.8% were isolated from urine culture, 5.6% from blood cultures, and the remaining isolates from a range of other clinical specimens (Table 2).

**TABLE 2:** *E. coli* clinical isolates by specimen type

Specimen type	n (%) of 250
Urine	207 (82.8%)
Blood	14 (5.6%)
Swab	9 (3.6%)
Sputum/tracheal aspirate	5 (2.0%)
Tissue	2 (0.8%)
Synovial fluid	1 (0.4%)
Other bodilyfluid	12 (4.8%)

All 250 isolates screened positive for AmpC by MASTDISC, and two isolates also screened positive for ESBL by this method. Seventy-two (28.8%) of all included isolates were determined to carry an acquired AmpC gene by PCR with 46 (18.4%) DHA positive, 25 (10.0%) CIT positive, and 1 isolate positive for both genes. A total of 170 isolates were additionally characterized by WGS. Among sequenced isolates, 82.9% (141/170) were AmpC negative, 14.1% (24/170) were positive for DHA, 2.4% (4/170) were positive for CIT, and 1 isolate was positive for both DHA and CIT. The rate of concordance between the 170 isolates that underwent WGS results and AmpC PCR results was 100%. No ESBL genes were detected in any isolates that underwent WGS, including from one of two isolates that were ESBL and AmpC screen positive on MASTDISC testing. The second isolate did not undergo WGS and was also PCR negative for AmpC gene detection.

### Comparison of phenotypic category and PCR result

Isolates with a positive AmpC screen were further phenotypically categorized by susceptibility to ceftazidime and ceftriaxone. Of these, 81 isolates (32.4%) were susceptible to both agents. A further 127 isolates (50.8%) were non-susceptible to only ceftazidime (MIC > 1 mg/L). The remaining 42 isolates (16.8%) were also non-susceptible to ceftriaxone (MIC > 1 mg/L). All ceftriaxone non-susceptible isolates were also non-susceptible to ceftazidime.

As shown in Table 3, increasing MICs to ceftazidime and ceftriaxone were associated with higher detection rates of AmpC genes. However, even the highest level of ceftriaxone resistance detected by our automated susceptibility platform (MIC > 4) did not alone reliably correlate with the presence of acquired AmpC genes. Similarly, the presence of susceptible MIC, ≤1 mg/L, did not rule out acquired AmpC gene carriage. DHA genes were significantly more likely than CIT genes to be detected in isolates with MIC ≤ 4 mg/L for both ceftazidime (*P* = 0.003) and ceftriaxone (*P* < 0.001).

**TABLE 3:** Correlation of ceftazidime and ceftriaxone MIC with AmpC PCR in *E. coli* isolates with a positive AmpC screening test^*a*^

Agent tested and MIC	Total *n*	AmpC gene detected
		DHA gene detected
		CIT gene detected
Ceftazidime MIC ≤ 1 mg/L	81	**3** (**3.7%**)
		DHA 2 (2.5%)
		CIT 1 (1.2%)
Ceftazidime MIC 2–4 mg/L	110	**29** (**26.3%**)
		DHA 24 (21.8%)
		CIT 4 (3.6%)
Ceftazidime MIC > 4 mg/L	59	**40** (**67.8%**)
		DHA 20 (33.9%)
		CIT 20 (33.9%)
Ceftriaxone MIC ≤ 1 mg/L	208	**41** (**19.7%**)
		DHA 39 (18.8%)
		CIT 2 (1%)
Ceftriaxone MIC 2–4 mg/L	13	**5** (**38.5%**)
		DHA 3 (23.1%)
		CIT 2 (15.4%)
Ceftriaxone MIC > 4 mg/L	29	**25** (**86.2%**)
		DHA 4 (13.7%)
		CIT 21 (72/4%)

^*a*^ Bolded entries represent the composite results of both DHA and CIT genes combined (i.e., the top Category of Amp C gene detected).

We further categorized *E. coli* isolates with a positive AmpC screen by two different phenotypic screening criteria (Materials and Methods, Table 1) which involve a higher threshold of cefoxitin resistance in addition to criteria for increased ceftazidime or ceftriaxone MIC. Using these criteria, 160 isolates (64%) met Criteria 1 and 26 isolates (10.4%) met Criteria 2.

Of isolates that met Criteria 1, 93/160 (58.1%) were PCR negative and 67/160 (41.8%) were PCR positive. Among isolates that did not meet Criteria 1, 85/90 (94.4%) were PCR negative, and 5/90 (5.5%) were PCR positive. When used to predict the carriage of an acquired AmpC gene using PCR, this phenotypic confirmatory method had a sensitivity of 93.1%, specificity of 47.8%, positive predictive value of 41.9%, and negative predictive value of 94.4% (Table 4). A total of 26 isolates met Criteria 2 for phenotypic confirmation. Of those that met Criteria 2, 2/26 (7.7%) were PCR negative and 24/26 (92.3%) were PCR positive. When used to predict acquired gene carriage result (AmpC PCR), the sensitivity was 33.3%, specificity was 98.9%, positive predictive value was 92.3%, and negative predictive value was 78.6% (Table 4).

**TABLE 4:** Phenotypic criteria 1 and 2 test evaluation with PCR confirmation

	Criteria 1	Criteria 2
Sensitivity	93.1%	33.3%
Specificity	47.8%	98.9%
Positive predictivevalue	41.9%	92.3%
Negative predictivevalue	94.4%	78.6%
Chi-square statistic (χ^2^)	37.1 (*P* < 0.001)	53.7 (*P* < 0.001)

## DISCUSSION

The detection of plasmid-mediated AmpC resistance in *E. coli* is challenging but remains important. As demonstrated in our cohort, the DHA family of genes is particularly likely to be present in isolates testing susceptible to ceftriaxone. Unlike most CIT family genes or intrinsic expression of *E. coli* BLA*_ampC_*, the DHA genes are inducible following treatment with beta-lactam antibiotics. Although poorly studied and, therefore, its incidence unknown, this phenomenon has been linked with clinical failures of treatment with third-generation cephalosporins (7, 17). Arguably, therefore, the detection of acquired AmpC genes important from both a clinical and infection control perspective. While numerous phenotypic and genotypic confirmatory tests have been proposed, an optimal method remains elusive. Furthermore, implementing these methods leads to increases in turnaround time, may be labor-intensive or require specialized equipment, and frequently come at a significant cost.

The finding of a link between high-level resistance to third- generation cephalosporins and plasmid-mediated AmpC β-lactamase production is consistent with the results of previous studies. As has been described by Edquist et al., nearly all isolates with plasmid-mediated AmpC enzymes demonstrated non-susceptibility to cefotaxime or ceftazidime (18). This was confirmed by our study with ceftazidime susceptibility present in only 3.7% of isolates with plasmid-mediated AmpC gene detection. By contrast, ceftriaxone susceptibility remained relatively common in isolates with acquired AmpC genes, with 58% of PCR positive isolates in our cohort testing susceptible to this antibiotic. Aarestrup et al. evaluated the performance of eight different cephalosporins in detecting cephalosporin resistance and found that ceftriaxone exhibited some capacity to discriminate between isolates with plasmid-mediated AmpC or ESBL enzymes and de-repressed chromosomal AmpC producers (19). Coolen et al. subsequently used machine learning to develop an algorithm for plasmid-mediated AmpC detection in cefoxitin-resistant *E. coli* isolates and found that a cefotaxime MIC > 6 mg/L could be used to discriminate between plasmid-mediated AmpC and de-repressed chromosomal AmpC producers (20). However, as our susceptibility panels do not include cefotaxime, we could not evaluate this as a potential phenotypic confirmatory test in our laboratory.

This study evaluated the performance of two proposed criteria sets for phenotypic AmpC confirmation based on antimicrobial susceptibility testing, and the results highlight some of the challenges associated with AmpC detection. Criteria 1 demonstrated high sensitivity but poor specificity, therefore performing poorly as a confirmatory test. However, this test had a high negative predictive value, meaning it was a useful test for ruling out plasmid-mediated AmpC gene carriage. These criteria could be utilized in settings where there is no ability to confirm phenotypic results with molecular testing, such as in resource-limited settings. In our laboratory, this criterion was used to ensure acquired AmpC resistance was not missed during the COVID pandemic when PCR resources were prioritized for viral testing.

By contrast, Criteria 2 demonstrated low sensitivity but high specificity for plasmid-mediated AmpC β-lactamases and is, therefore, a suitable test for phenotypic confirmation. However, few isolates met these relatively stringent criteria (10.4%), leaving the vast majority of remaining screen positives needing to undergo a molecular test to confirm the presence of an acquired AmpC gene, as is currently recommended by the EUCAST antimicrobial susceptibility testing guidelines (10).

Depending on factors such as availability and cost-effectiveness, another potential strategy is to screen all isolates with a molecular test and bypass phenotypic testing entirely. Whil only a subset of isolates were available for WGS, there was complete concordance between our PCR and WGS results among those tested in this study, confirming the previous finding that DHA and CIT genes predominate in the region serviced by our laboratory. This finding provides further evidence that targeted HRM PCR is a reliable method of AmpC β-lactamase detection, provided that WGS is performed periodically to establish the local circulating genes to target in molecular assays.

In our laboratory, the findings of this study have led to an alteration in the AmpC detection algorithm. Currently, the protocol involves screening Enterobacterales isolates with the criteria of a cefoxitin MIC ≥8 mg/L and a positive MASTDISC D68C test. Following screening, if isolates meet Criteria 2 (cefoxitin MIC > 16 mg/L and ceftriaxone MIC > 4 mg/L), they are reported as phenotypically confirmed AmpC β-lactamase producers. Isolates that have a positive screen but do not meet phenotypic confirmatory criteria then proceed to molecular testing with the HRM PCR described here. A limitation of this study is that it specifically evaluated phenotypic confirmatory testing in a pre-screened population therefore, findings are unable to be applied to wild-type isolates. Neither set of phenotypic criteria was both specific and sensitive for AmpC production, supporting utilization of a two-step process incorporating an initial screening test, followed by a confirmatory test. Furthermore, isolates used in this study consisted of only *E. coli* isolates, hence the findings of this study are unable to be extrapolated to other organisms. Additionally, the performance of antimicrobial susceptibility testing in predicting plasmid-mediated AmpC gene expression is dependent on the specific genetic families that are involved, hence geographic variation in the distribution of genes may limit the application of these findings to isolates in other locations.

In conclusion, the detection of plasmid-mediated AmpC genes remains challenging as the expression of an AmpC phenotype remains common in *E. coli* without acquired resistance. Molecular confirmation of plasmid-mediated AmpC genes remains the gold standard, with complete concordance between the HRM PCR approach we describe here and WGS. The phenotypic criteria described outlined in this study may serve as a relatively simple, inexpensive, but less accurate methods of determining the likelihood of plasmid-mediated AmpC carriage in a pre-screened population of *E. coli* isolates. Ongoing surveillance of resistance genes in a population with WGS is advised to monitor the emergence and regional distribution of plasmid-mediated AmpC genes.

## SUPPLEMENTAL MATERIAL

Supplemental methods spectrum.00933-24-s0001.docx PCR development methods.

ASM does not own the copyrights to Supplemental Material that may be linked to, or accessed through, an article. The authors have granted ASM a non-exclusive, world-wide license to publish the Supplemental Material files. Please contact the corresponding author directly for reuse.

## References

[1] Antimicrobial Resistance Collaborators. 2022. Global burden of bacterial antimicrobial resistance in 2019: a systematic analysis. Lancet 399:629–655. doi:10.1016/S0140-6736(21)02724-0 35065702 PMC8841637

[2] Jacoby GA. 2009. AmpC β-lactamases. Clin Microbiol Rev 22:161–182. doi:10.1128/CMR.00036-08 19136439 PMC2620637

[3] Doi Y, Paterson DL. 2007. Detection of plasmid-mediated class C β-lactamases. Int J Infect Dis 11:191–197. doi:10.1016/j.ijid.2006.07.008 17339123

[4] Tamma PD, Doi Y, Bonomo RA, Johnson JK, Simner PJ, Antibacterial Resistance Leadership Group. 2019. A primer on AmpC β-lactamases: necessary knowledge for an increasingly multidrug-resistant world. Clin Infect Dis 69:1446–1455. doi:10.1093/cid/ciz173 30838380 PMC6763639

[5] Wachino J, Kurokawa H, Suzuki S, Yamane K, Shibata N, Kimura K, Ike Y, Arakawa Y. 2006. Horizontal transfer of bla_CMY_-bearing plasmids among clinical Escherichia coli and Klebsiella pneumoniae isolates and emergence of cefepime-hydrolyzing CMY-19. Antimicrob Agents Chemother 50:534–541. doi:10.1128/AAC.50.2.534-541.2006 16436707 PMC1366887

[6] Pérez-Pérez FJ, Hanson ND. 2002. Detection of plasmid-mediated AmpC beta-lactamase genes in clinical isolates by using multiplex PCR. J Clin Microbiol 40:2153–2162. doi:10.1128/JCM.40.6.2153-2162.2002 12037080 PMC130804

[7] Papanicolas LE, Bell JM, Weldhagen GF, Bastian I. 2013. Ceftriaxone treatment failure in cephalosporin-susceptible Escherichia coli bacteraemia. Int J Antimicrob Agents 41:298–299. doi:10.1016/j.ijantimicag.2012.11.008 23312600

[8] Peter-Getzlaff S, Polsfuss S, Poledica M, Hombach M, Giger J, Böttger EC, Zbinden R, Bloemberg GV. 2011. Detection of AmpC beta-lactamase in Escherichia coli: comparison of three phenotypic confirmation assays and genetic analysis. J Clin Microbiol 49:2924–2932. doi:10.1128/JCM.00091-11 21653764 PMC3147754

[9] CLSI. 2018. Performance standards for antimicrobial susceptibility testing. In CLSI supplement M100, 28th ed. Clinical and laboratory standards institute, Wayne, PA.

[10] The European Committee on Antimicrobial Susceptibility Testing. 2017. EUCAST guideline for the detection of resistance mechanisms and specific resistances of clinical and/or epidemiological importance. Available from: https://www.eucast.org/resistance_mechanisms

[11] Tagg KA, Ginn AN, Jiang X, Ellem J, Partridge SR, Iredell JR. 2015. Distribution of acquired AmpC β-lactamase genes in Sydney, Australia. Diagn Microbiol Infect Dis 83:56–58. doi:10.1016/j.diagmicrobio.2015.06.001 26099646

[12] Tamma PD, Sharara SL, Pana ZD, Amoah J, Fisher SL, Tekle T, Doi Y, Simner PJ. 2019. Molecular epidemiology of ceftriaxone non-susceptible Enterobacterales isolates in an academic medical center in the United States. Open Forum Infect Dis 6:fz353. doi:10.1093/ofid/ofz353 PMC6736082 31401649

[13] Harris PNA, Ben Zakour NL, Roberts LW, Wailan AM, Zowawi HM, Tambyah PA, Lye DC, Jureen R, Lee TH, Yin M, Izharuddin E, Looke D, Runnegar N, Rogers B, Bhally H, Crowe A, Schembri MA, Beatson SA, Paterson DL, MERINO Trial investigators. 2018. Whole genome analysis of cephalosporin-resistant Escherichia coli from bloodstream infections in Australia, New Zealand and Singapore: high prevalence of CMY-2 producers and ST131 carrying bla_CTX-M-15_ and bla_CTX-M-27_. J Antimicrob Chemother 73:634–642. doi:10.1093/jac/dkx466 29253152

[14] The European Committee on Antimicrobial Susceptibility Testing. 2024. Clinical breakpoints (v 14.0). Available from: https://www.eucast.org/clinical_breakpoints

[15] Geyer CN, Hanson ND. 2014. Multiplex high-resolution melting analysis as a diagnostic tool for detection of plasmid-mediated AmpC β-lactamase genes. J Clin Microbiol 52:1262–1265. doi:10.1128/JCM.00214-14 24478414 PMC3993522

[16] Alcock BP, Huynh W, Chalil R, Smith KW, Raphenya AR, Wlodarski MA, Edalatmand A, Petkau A, Syed SA, Tsang KK, et al.. 2023. CARD 2023: expanded curation, support for machine learning, and resistome prediction at the comprehensive antibiotic resistance database. Nucleic Acids Res 51:D690–D699. doi:10.1093/nar/gkac920 36263822 PMC9825576

[17] Pai H, Kang C-I, Byeon J-H, Lee K-D, Park WB, Kim H-B, Kim E-C, Oh M-D, Choe K-W. 2004. Epidemiology and clinical features of bloodstream infections caused by AmpC-type-β-lactamase-producing Klebsiella pneumoniae. Antimicrob Agents Chemother 48:3720–3728. doi:10.1128/AAC.48.10.3720-3728.2004 15388426 PMC521917

[18] Edquist P, Ringman M, Liljequist BO, Wisell KT, Giske CG. 2013. Phenotypic detection of plasmid-acquired AmpC in Escherichia coli—evaluation of screening criteria and performance of two commercial methods for the phenotypic confirmation of AmpC production. Eur J Clin Microbiol Infect Dis 32:1205–1210. doi:10.1007/s10096-013-1869-x 23549664

[19] Aarestrup FM, Hasman H, Veldman K, Mevius D. 2010. Evaluation of eight different cephalosporins for detection of cephalosporin resistance in Salmonella enterica and Escherichia coli. Microb Drug Resist 16:253–261. doi:10.1089/mdr.2010.0036 20624078

[20] Coolen JPM, den Drijver EPM, Kluytmans JAJW, Verweij JJ, Lamberts BA, Soer JACJ, Verhulst C, Wertheim HFL, Kolwijck E. 2019. Development of an algorithm to discriminate between plasmid- and chromosomal-mediated AmpC β-lactamase production in Escherichia coli by elaborate phenotypic and genotypic characterization. J Antimicrob Chemother 74:3481–3488. doi:10.1093/jac/dkz362 31504559 PMC7183348

