# Supplementary material for: Characterizing Escherichia coli carrying plasmid-mediated AmpC β-lactamases to optimize detection in a diagnostic laboratory setting
Source: Microbiol Spectr. 2024 Nov 7;12(12):e00933-24. doi: 10.1128/spectrum.00933-24 (PMC11619294; doi:10.1128/spectrum.00933-24)
Supplement: Supplemental methods — PCR development methods. [file spectrum.00933-24-s0001.docx]

Characterising Escherichia coli Carrying Plasmid-Mediated AmpC β-Lactamases to Optimise Detection in a Diagnostic Laboratory Setting.

**Supplementary Information:**

The published protocol by Geyer described a multiplex PCR with high resolution melt to distinguish between MOX, FOX, ACC, DHA, CMY-2 (CIT), and ACT. This protocol was initially evaluated at SA Pathology and was observed that melt analysis did not provide a sufficient level of discrimination to accurately differentiate different AmpC enzymes from control organisms in our possession.

After some discussion we decided to simplify the protocol to identify and discriminate between DHA and CMY type AmpC enzymes only. These AmpC groups are the most clinically important AmpCs we see in our patient population and accounted for all AmpCs detected by WGS during the first half of 2021 (with the exception of chromosomal enzymes in *Enterobacter cloacae*). Primers for MOX, FOX, ACC, and ACT were omitted from the final assay.

To automate the process of reporting AmpC results based on the crossing point and melt analysis of amplified products, an algorithm was written to determine the AmpC result and type based on crossing point and melt temperature.

All AmpC-Detected samples prepared as described resulted in Cq values between 12 - 20. CMY-positive samples exhibited melts ranging between 84.8 and 85.1, whilst DHA-positive samples exhibited higher melts ranging between 86.0 and 86.3. These findings were utilized to determine the cut-off values of an algorithm, which is automated via a Microsoft Excel script in a worksheet and automates the process of reporting both the AmpC result and the type of enzyme identified.

**AmpC Not Detected definition:**

- If Cp >30
- If Melt curve is not present
- If Cp <=30, but melt <84 ^o^c

**AmpC Detected definition:**

- If Cp <=30 and melt is >=84 ^o^c

**If AmpC Detected (Cp <=30 and melt is >=84** ^o^c**):**

- Cp <85.8, report CIT
- Cp >=85.8, report DHA

Very rarely a melt will be observed that will fall outside of the normal temperature range of controls. If an outlier is observed, the AmpC Type Interpretation field is manually changed to report “Not Determined”.


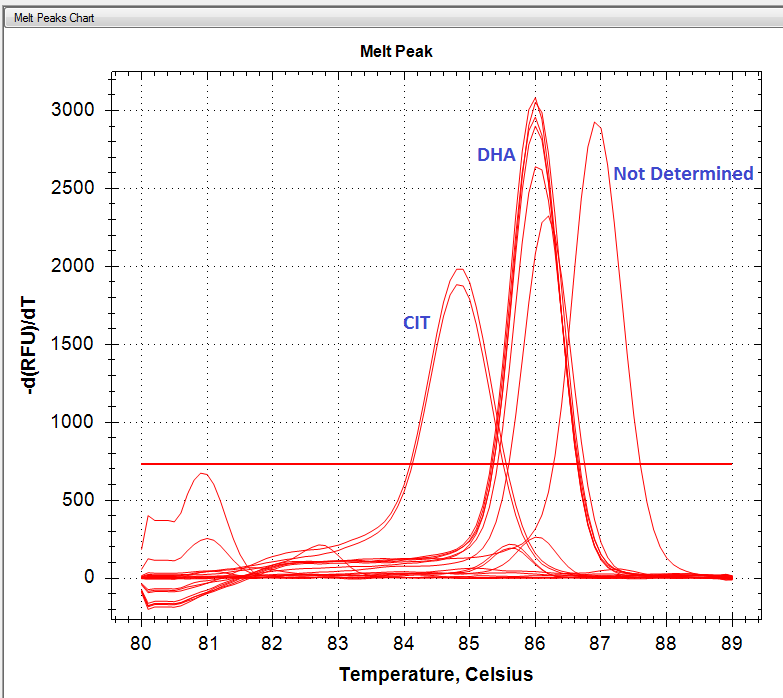


**Primer Sequences**

DHA-F: 5’- AACTTTCACAGGTGTGCTGGGT-3’

DHA-R: 5’- TCAGCAGATCCGCACGGCTT -3’

CMY2-F: 5’- TCCAGCGTTATTGATATGG -3’

CMY2-R: 5’- CATCTCCCAGCCTAATCC -3’

###### DHA Primer Binding Site (197 bp amplicon)


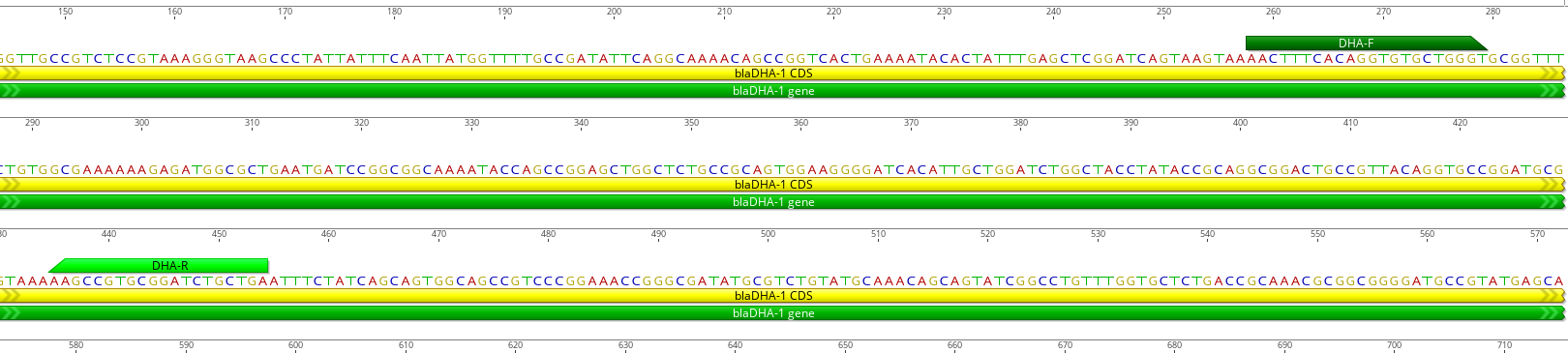


**CMY2 Primer Binding Site (147 bp amplicon)**


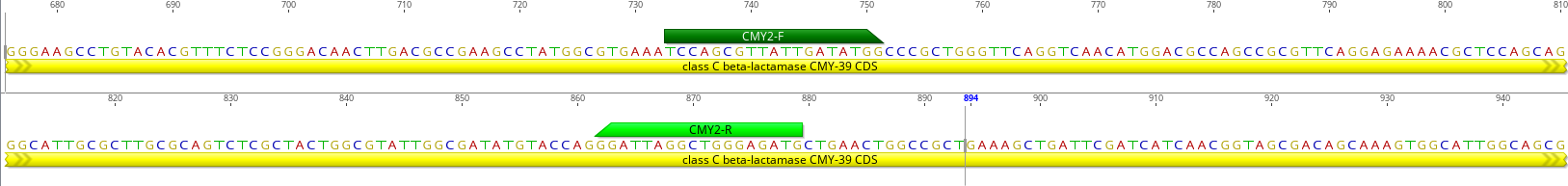


**Master Mix Preparation**

| **Reagent** | **Volume μL/test** | **Final Conc.** | **Volume (μL)** |
| --- | --- | --- | --- |
| **EpiTect Type-iT HRM PCR Buffer** | 12.50 | 1X | **12.50** |
| **CMY2-F(100μM)** | 0.125 | 0.5μM | **0.13** |
| **CMY2-R (100μM)** | 0.125 | 0.5μM | **0.13** |
| **DHA-F(100μM)** | 0.125 | 0.5μM | **0.13** |
| **DHA-R (100μM)** | 0.125 | 0.5μM | **0.13** |
| **Water** | 9.00 |  | **9.00** |
| **Total MMX** | 22.00 |  | **22.00** |
| **Sample** | 3 |  |  |
| **TOTAL** | **25.00** |  |  |

**Cycling Parameters**

AmpC PCR thermal profile, lid heated:

| **Step** | **Temperature** | **Duration** |
| --- | --- | --- |
| 1. Initial Denaturation | 95°C | 5 minutes |
| 2. Denaturation | 95°C | 10 seconds |
| 3. Annealing | 55°C | 30 seconds |
| 4. Extension and Acquisition | 72°C | 10 seconds |
| 5. Goto 2, 39 times ( total 40 cycles) | | |
| 6. High Resolution Melt | 80°C - 89°C | + 0.1°C / cycle |
|  |  | Ramp 0.1°C / second with acquisition |

Specificity

112 *Enterobacteriaceae* isolates in which WGS did not identify the presence of AmpC genes were evaluated using the AmpC HRM NAT (110 *Escherichia coli*, 1 *Klebsiella pneumonia*, 1 *Klebsiella aerogenes*).

Following testing with the AmpC HRM NAT and applying the reporting algorithm:

- 112 WGS AmpC Not Detected samples resulted in 111 AmpC HRM NAT Not Detected and *1 AmpC HRM NAT Detected sample.

*This isolate was identified as *Klebsiella aerogenes* and produced a PCR positive result with Cq 26.98 and melt 87.4. Interpreting the melt as an outlier, the sample was manually reported as ‘Not Determined’. The enzyme was not identified via WGS, however *K. aerogenes* is known to harbor intrinsic AmpC which explains the result.
